# The hidden wolf – Case report of valproic acid drug-induced cerebral lupus erythematosus

**DOI:** 10.1192/j.eurpsy.2025.823

**Published:** 2025-08-26

**Authors:** S. C. Steininger, R. Mansky, A. Reichert, K. Brändle, S. Bacanovic, W. Kawohl, F. Xepapadakos

**Affiliations:** 1 Old Age Psychiatry, Clienia Schloessli, Private Psychiatric Hospital and Academic Teaching Hospital of the University of Zurich and of the University of Nicosia Medical School, Oetwil am See/Zurich; 2 Radiology, Männedorf Hospital, Männedorf; 3 Clienia Schloessli, Private Psychiatric Hospital and Academic Teaching Hospital of the University of Zurich and of the University of Nicosia Medical School, Oetwil am See/Zurich, Switzerland; 4 Department of Basic and Clinical Sciences at the University of Nicosia Medical School, Nicosia, Cyprus

## Abstract

**Introduction:**

Neuropsychiatric symptoms associated with valproic acid were already described in the 1980s. Valproic acid is known to cause valproate encephalopathy and hyperammonemia^(1,2)^. Rarely it can cause drug induced lupus erythematosus^(5)^. Previous studies and case reports have documented manifestations such as rash, pulmonary involvement, pleuritis and carditis due to valproate-induced lupus erythematosus. However, little is known about other manifestations^(3,4,6)^. To date, there are no descriptions of cerebral drug-induced lupus erythematosus due to valproic acid.

**Objectives:**

We report the case of a female 63-year-old patient who was initially treated in our inpatient clinic for prolonged delirium and major neurocognitive disorder following severe traumatic brain injury with bilateral traumatic intracerebral temporal bleeding eight months prior. Symptoms from the severe traumatic brain injury included aphasia, impulse control disorder, reduced frustration tolerance and depression, which were treated with valproic acid and quetiapine. The patient experienced a strongly fluctuating, progressively deteriorating neuropsychiatric syndrome that began six months before hospitalization. Additionally, a fluctuating neurological syndrome including temporary complete hemiparesis, hyperreflexia and loss of consciousness, which lasted from 30min to hours, and halluzinations was observed. The patient also developed epileptic seizures, which could not be managed by a combined antiepileptic therapy with valproic acid, brivaracetam and clobazam. Previous examinations (brain-MRI and CT, CFS, extended lab testing) excluded acute cerebrovascular insult, encephalitis and hyperammonemia.

**Methods:**

The diagnosis of cerebral vasculitis was considered after excluding infection or cerebrovascular insult. Anti-nuclear antibodies and anti-histone Antibodies were detected in the blood sample. Anti-NMDA receptor antibodies as well as antibodies for paraneoplastic syndromes and Bickerstaff encephalitis were not detected. MRI scans over eight months showed an increase of white matter lesions periventricular and in the brain stem.

**Results:**

Based on these results and the medical history, we considered a drug-induced vascular lupus erythematosus due to valproic acid. We initiated immunosuppressive therapy with high-dose prednisone while tapering valproate acid. Within days, the neurological symptoms declined and epileptic seizures ceased.

**Image 1:**

**Figure:**
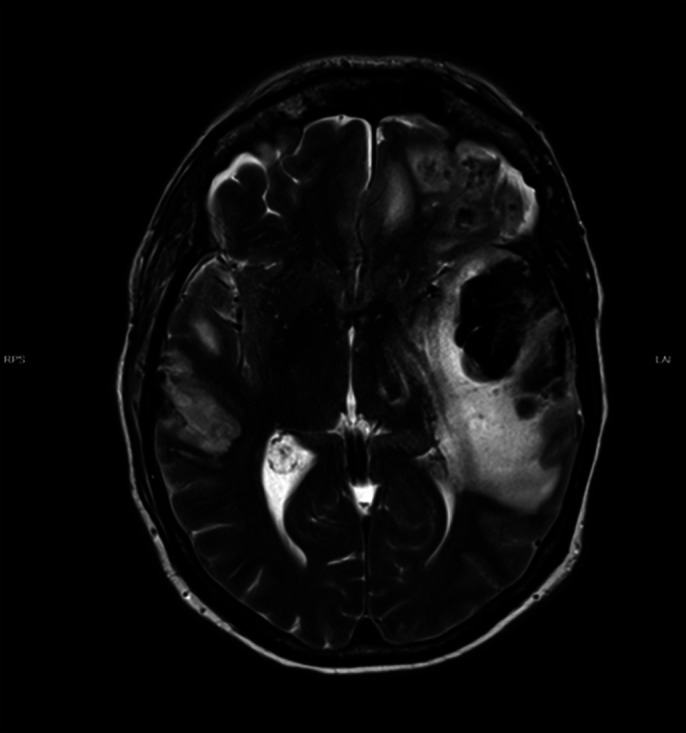

**Image 2:**

**Figure:**
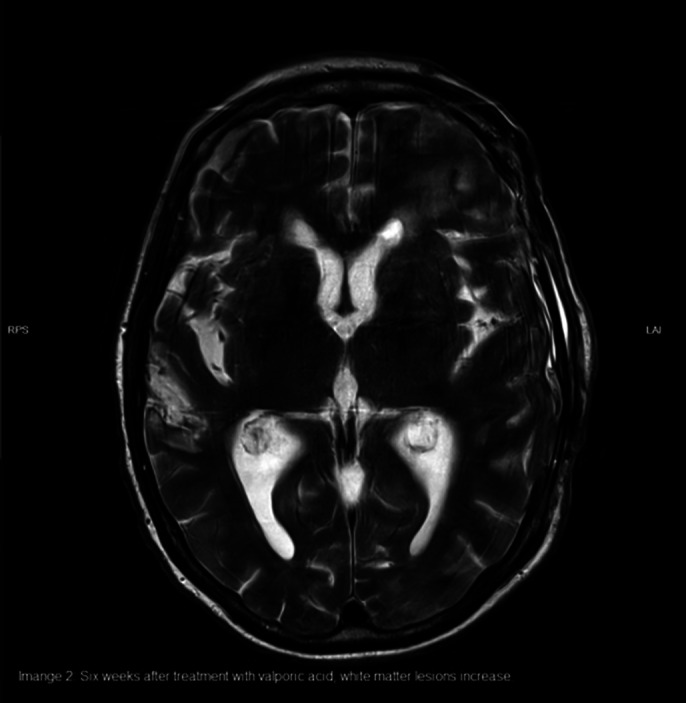

**Image 3:**

**Figure:**
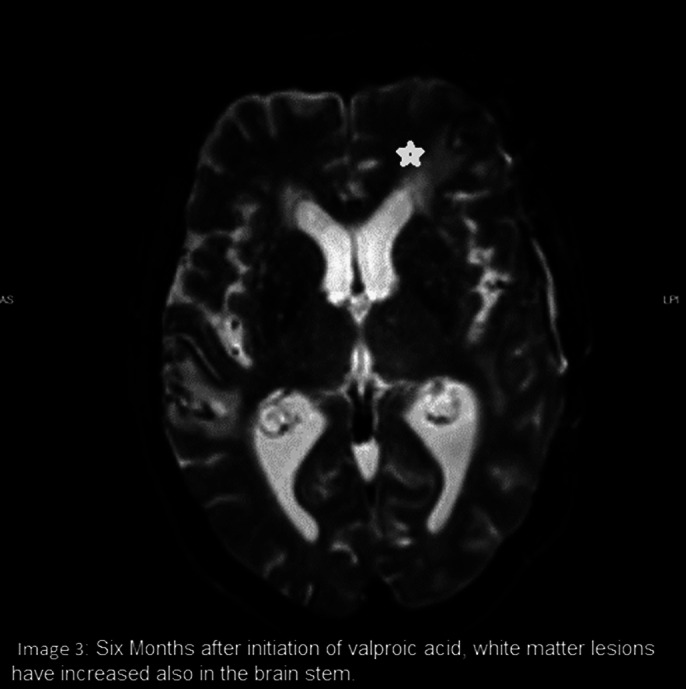

**Conclusions:**

This is the first described case of valproic acid-induced cerebral lupus erythematosus. There are no established recommendations for therapy or knowledge about the course and outcome of this condition. This case highlights the importance of evaluating valproate-induced lupus erythematosus in patients with fluctuating neuropsychiatric symptoms under valproic acid medication, in addition to valproate-induced encephalopathy. Prednisone might be a viable treatment option.

**Disclosure of Interest:**

None Declared

